# Factors affecting development of *Clostridium difficile* infection in hospitalized pediatric patients in the country Georgia

**DOI:** 10.1186/s13104-018-3517-0

**Published:** 2018-06-26

**Authors:** Iuri Migriauli, Vakhtang Meunargia, Ivane Chkhaidze, Giorgi Sabakhtarishvili, Kakha Gujabidze, Maia Butsashvili, George Kamkamidze

**Affiliations:** 1 AIETI Medical School, David Tvildiani Medical University, Tbilisi, Georgia; 2 M. Iashvili Central Children Hospital, Tbilisi, Georgia; 3 Tbilisi State Medical University, Tbilisi, Georgia; 4 Health Research Union and Clinic NeoLab, Tbilisi, Georgia

**Keywords:** *Clostridium difficile*, Hospitalized children, Antibiotics, EIA, PCR

## Abstract

**Objective:**

Main aims of our study were to investigate occurrence of *Clostridium difficile* among hospitalized pediatric patients in Georgia and examine risk factors for the development of *C. difficile* infection. During our study we tested and piloted the real-time PCR diagnostic systems for rapid and simultaneous identification of *C. difficile* and number of other pathogens in our facility settings. A cross-sectional study has been performed in children less than 18 years of age in two pediatric hospitals in Georgia, between May 2016 and December 2017. Stool specimens negative by the conventional bacteriology analysis were analyzed for the presence of *C. difficile* and several viral and protozoa pathogens using enzyme immune assay and polymerase chain reaction. In total samples from 220 hospitalized children with gastroenteritis symptoms were analyzed in this study.

**Results:**

The average age of the study participants was 4.7 years. Overall 23 children were identified positive for *C. difficile* (10.5%). Antibiotic exposure within 2 months preceding the onset of diarrhea was associated with an increased risk of *C. difficile* infections. The risk was greatest with cephalosporins, followed by penicillins, carbapenems and macrolides. *Clostridium difficile* is an important cause of healthcare-associated diarrhea in pediatric population of Georgia.

## Introduction

*Clostridium difficile*, a Gram-positive, spore-forming bacillus is transmitted by fecal–oral route or from environment [1]. *Clostridium difficile* infections occurrence has been increased among hospitalized pediatric patients across the world [2]. Colitis associated with *C. difficile* is the result of a derangement of normal bacterial colonic flora. *Clostridium difficile* colonization causes release of enterotoxins and cytotoxins, which cause diarrhea, inflammation and damage of mucosa [3]. Two different toxins are made by pathogenic strains of *C. difficile*. Toxin A, is an enterotoxin and toxin B, a cytotoxin. Both of these toxins are high-molecular weight proteins, which bind specific receptors on the intestinal mucosal cells [3].

Clinical manifestations of *C. difficile* vary from asymptomatic colonization to pseudomembranous colitis with signs of fever, severe abdominal pain and bloody diarrhea [2]. Even short-term use of a single antibiotic can potentially cause *C. difficile* colitis. Risk of colitis increases, if two or more antibiotics are used, or antibiotic course is prolonged [3].

*Clostridium difficile* infections are increasing among hospitalized pediatric patient [2]. Recent articles of clinical practice guideline for infections caused by *C. difficile* in adult patients didn’t encompass issues that are specific to pediatric patients, because previously it was believed that *C. difficile* infections were more common in elderly patients and specific populations such as children and young adults were at low risk [2, 3]. Diagnosis of *C. difficile* associated colitis should be suspected in all hospitalized patients having diarrhea [3]. Available stool assays to diagnose *C. difficile* infection are: stool culture, stool cytotoxin test, enzyme linked immunoassay (EIA), real-time polymerase chain reaction (PCR) assay, EIA to detect toxins A and B and Latex agglutination test [3]. At this time, the most commonly used test to detect *C. difficile* toxins is commercially available EIA, which can detect toxin A and/or toxin B in stool samples [2]. In recent studies, the sensitivity of real-time PCR for toxins A and B compared with EIA for toxins A and B were superior and the specificity was equal. The number of repeated samples decreased with the use of the PCR, which is currently a preferred and approved method for diagnosis of *C. difficile* infection by many hospitals and laboratories, but more studies are needed before PCR can be used routinely [2].

The aim of our study was to examine risk factors for *C. difficile* infection and evaluate occurrence of *C. difficile* and some other gastrointestinal pathogens among hospitalized pediatric patients in Georgia.

The definition of pediatric patients is children aged from 1 month of age to 18 years. Diarrhea in pediatric patients by The World Health Organization is defined as “the passage of three or more loose or liquid stools per day, or more frequently than is normal for the individual” [4–7]. The definition of antibiotic associated diarrhea is diarrhea associated with antibiotic use, either during antibiotic exposure or for up to 8 weeks after discontinuation of antibiotic therapy [4, 8, 9]. Although etiologic agents for antibiotic associated diarrhea vary and sometimes pathogens can’t be identified, up to one-third of antibiotic associated diarrhea cases are caused by *C. difficile* infection. Etiologies of antibiotic associated diarrhea among pediatric patients may also include viruses (20%) [4, 10] or *C. difficile* (25%), but also may be caused directly by osmotic imbalances in the intestines due to antibiotic exposure and disruption of normal bacterial flora.

Gender doesn’t seem to have any significant impact on *C. difficile* infection in pediatric patients, as the incidence of these cases among female and male patients is nearly equivalent [4]. Risk factors predisposing to antibiotic associated diarrhea can be divided into two main groups: host factors (e.g., age) and disruptive factors (e.g., antibiotics) that may have damaging effect on the normally protective intestinal bacterial flora [4, 9, 11]. The incidence of antibiotic associated diarrhea may be even higher (65%) if broad-spectrum antibiotics (cephalosporins, penicillins, carbapenems, macrolides, etc.) were used [4]. The number of clinical studies determining and identifying risk factors for antibiotic associated diarrhea in children is limited.

Therefore, the aim of this study was to determine risk factors for hospitalized children during the incident *C. difficile* infection episode that are associated with developing *C. difficile* infection within 60 days during hospitalization or after discharge and to evaluate risks related with the specific antibiotics in order to better understand the risks of prescribing various antibiotics in the community and hospital setting.

## Main text

### Methods

A cross-sectional study was performed in children < 18 years of age in two pediatric hospitals between May 2016 and December 2017. Stool samples were collected from the pediatric patients admitted to two large tertiary children’s hospitals in Tbilisi, capital of Georgia. Hospitals involved in the study were: M. Iashvili Children’s Central Hospital and Tbilisi Children’s Clinical Hospital for Infectious Diseases. Both hospitals are admitting patients from capital and patients transferred from other regional hospitals around Georgia. Laboratory investigations were done in the Clinic NeoLab, Tbilisi, Georgia.

Stool specimens were analyzed for the presence of *C. difficile* using a two-step testing algorithm including PCR and EIA. Totally 220 children meeting the definitions of this investigation who met our inclusion criteria were enrolled in this study.

*Clostridium difficile* infection was defined as the presence of any gastrointestinal symptoms accompanied by a clinical suspicion of *C. difficile* infection as well as a positive result for *C. difficile* toxins from rapid stool testing or *C. difficile* confirmation by PCR method or both. Final determinations were carried out by the attending physician or the hospital’s infection control team [12].

Stool of patients negative by the conventional bacteriology analysis covering enteric bacterial pathogens was investigated. Enzyme immunoassay testing kits for *C. difficile* toxins A and B were used as the rapid testing method (CerTestbiotec, Zaragoza, Spain). EIA test positive samples were analyzed by multiplex real-time PCR using Neo_PCR_Diagnostics system (NeoLab, Tbilisi, Georgia) [13] for confirmation of the infection and for simultaneous identification of additional gastrointestinal pathogens including *Entamoeba histolytica*, *Giardia lamblia*, *Cryptosporidium parvum, Adenovirus, Rotavirus, Norovirus* and *Astrovirus*. DNA extraction from the stool samples was performed by DNA purification system (OxGEn Molecular Solutions, Tbilisi, Georgia) [13]. *Clostridium difficile* EIA negative samples were tested for the presence of the above listed pathogenic agents using the same type EIA kits as for *C. difficile* described above (CerTestbiotec, Zaragoza, Spain) for presence of the corresponding pathogen.

The sample size calculation was performed for Chi Squared Statistic to compare proportions of dichotomous variables for the following parameters: α = 0.05 (two-sided), β = 0.20, proportion of subjects with the outcome in one of the groups = 10%, odds ratio (OR) ≥ 2.0 [14]. By these calculations the minimum number of patients who had to be included in the study was equal to 219. Patient data were extracted from their medical charts and entered into the MS Excel spreadsheet. IBM SPSSV.21 software was used for the statistical analysis. The factors associated with the *C. difficile* infection were categorized into dichotomous variables and Pearson’s Chi square test was applied to define statistical significance p. The p value < 0.05 was considered as statistically significant.

### Results

The average age of the study participants was 4.7 years. Overall 23 children were identified positive for *C. difficile* (10.5%).

In samples negative for *C. difficile* the following enteric pathogens have been revealed with the following frequencies: *Rotavirus* in 14 cases (6.4%), *Adenovirus* in 13 (5.9%), *Giardia lamblia* in 11 (5.0%), *Astrovirus* in 4 (1.8%), *Cryptosporidium parvum* in 4 (1.8%), *Entamoeba histolytica* in 3 (1.4%) and *Norovirus* in 3 (1.4%) cases.

Out of 23 *C. difficile* positive patients, in four children we have identified co-infection with *Giardia lamblia* (17.4% co-infection rate) and in three children we identified co-infection with *Adenovirus* (13% co-infection rate).

Agreement between the PCR-based and EIA-based tests was excellent having disagreement only for one case where PCR was positive for Adenovirus while negative for this virus by EIA.

The study of the association of several factors with the development of *C. difficile* infection showed (Table 1) that the age more than 4 years old represented independent risk-factors of the development of *C. difficile* infection, while the gender and ethnicity was not associated with the infection. Antibiotic exposure within the month preceding the onset of diarrhea was statistically significantly associated with an increased risk of *C. difficile* infections. The risk was greatest with cephalosporins, followed by penicillins, carbapenems and macrolides.

**Table 1:** Association of different factors with the development of *Clostridium difficile* infection

Factor	Total number	*Clostridium difficile* positive	Odds ratio (OR)	95% confidence interval (CI)	Statistical significance p
Age (years)					
<4	158	12 (7.59%)	1		
≥ 4	62	11 (17.74%)	2.61	1.06–6.39	< 0.05
Gender					
Male	112	13 (11.61%)	1.29	0.53–3.16	> 0.05
Female	108	10 (9.26%)	1		
Ethnicity					
Georgian	198	20 (10.10%)	1		
Other	22	3 (13.64%)	1.4	0.31–4.83	> 0.05
Antibiotic use					
Yes	165	21 (12.73%)	3.85	1.2–9.6	< 0.05
No	55	2 (3.64%)	1		

### Discussion

Over 19,000 cases of diarrheal diseases are reported annually through state registration system in Georgia [15]. Over 70% of all registered cases are among children and one-fifth of cases are reported in Tbilisi. In over 80% of in-patient cases of diarrheal diseases covered by the public system in 2015, specific causing agent was not identified, signifying the lack of access to proper diagnostics and limitation of targeted treatment [15].

Our study showed a substantial portion of the *C. difficile* infection among the pediatric patients whose stool samples were negative by conventional bacteriology investigations. In addition, diverse viral and protozoa pathogens—both as the independent agents and as a co-infection to the *C. difficile*, were detected in these patients. Study also showed independent association of age and use of antibiotics with development of *C. difficile* infection.

*Clostridium difficile* is the frequent pathogenic agent causing diarrheal disease among hospitalized pediatric patients. Development of *C. difficile* related diarrhea is associated with the antibiotic treatment of pediatric patients hospitalized due to the different clinical diagnosis.

In the substantial portion of cases the antibiotic treatment is not optimal leading to the prolonged diarrhea and activation of *C. difficile* infection which is difficult to manage. Our study also shows that besides *C. difficile* infection other pathogens including viruses and parasites are responsible for the development of gastrointestinal infection and these infections cannot be managed by antibiotic treatment.

Our data are in agreement with the results of studies performed by other research groups in different countries, indicating to the growing importance of the *C. difficile* infection in the gastrointestinal morbidity among children in the whole world.

Studies carried out by different research groups on pediatric antibiotic associated diarrhea have shown [4] that there is three main factors affecting development of *C. difficile* infection: (1) patient factors—age, gender, comorbidities; (2) factors damaging protective intestinal normal bacterial flora—surgery, nasogastric tube feeding, gastrostomy, antibiotics, different medications; (3) long-term exposure to *C. difficile* spores (longer hospital stays, previous admissions, staying with infected patients in same room) [4, 16, 17]. Regardless of the above listed factors, not all act equally in the pediatric and adult patients. The risk factors that are common for pediatric *C. difficile* infection include: age 1–4 years old, comorbidities (cancer patients and patients with inflammatory bowel disease), use of antibiotics within previous 8 weeks (especially several antibiotics at the same time, cephalosporins, penicilins and macrolids), and history with hospitalization [4, 18, 19].

The most significant risk factor for recurrent disease is antibiotic exposure, including the use of non-CDI agents during treatment of *C. difficile* infection. In addition, a history of *C. difficile* infection and underlying comorbidities, particularly those related to problems of immune system are associated with the recurrence of infection [1, 20, 21].

When individual is hospitalized or exposed to *C. difficile* spores, two factors play important role in development of *C. difficile* infection: disruption of the healthy, highly diverse colonic normal bacterial flora and no preformed *C. difficile* antitoxin antibodies and/or failure of proper humoral immune response development against *C. difficile* toxins [22]. Accordingly, *C. difficile* is classified by CDC as one of three antibiotic-resistant infections that “are immediate public health threats that require urgent and aggressive action” [22, 23]. Although pediatric patients have not previously been known to be at high-risk for *C. difficile* infection, recent studies indicate that *C. difficile* infection is currently increasing in pediatric patients in both community and hospital settings [24–26]. The rate of hospitalized children with *C. difficile* infection has nearly doubled in the last decade [24, 27].

*Clostridium difficile* is a one of the principal cause of antibiotic-associated diarrhea in pediatric patients. One factor that complicates the assessment of the role of *C. difficile* infection in children is the possibility of co-infection with other gastrointestinal microorganisms. In this study, we identified *C. difficile* co-infections in young children, in an effort to discuss the frequency of co-infections and their possible role in the *C. difficile* infection clinical presentation severity. The summed percentage of identified co-infections was 34.2%. However, the spectrum of co-infections tested differed significantly among studies and 38% of stated co-infections did not described any pathogen.

Small sample sizes and unclear *C. difficile* infection case definitions hinder meaningful conclusions on the true rate of co-infections in this patient population. According to this study, one can assume that co-infections may be common in children with diarrhea who had positive *C. difficile* test results. *Clostridium difficile* is a significant cause of hospital associated diarrhea in pediatric population of Georgia. The findings share similarities with the studies from other countries, establishing the administration of broad-spectrum antibiotics as a risk factor for *C. difficile* infections.

## Limitations

The number of clinical studies determining risk factors for pediatric antibiotic associated diarrhea is limited. In young children a broad panel of pathogens should be tested for to exclude other microbiological causes. However, small sample size in our study and poor quality of the available literature on this subject highlights a need for further studies.

## Abbreviations

CDI: *Clostridium difficile* infection
EIA: enzyme immune assay
PCR: polymerase chain reaction
WHO: World Health Organization
